# Evolution of factors shaping the endoplasmic reticulum

**DOI:** 10.1111/tra.12863

**Published:** 2022-08-17

**Authors:** Aspasia Kontou, Emily K. Herman, Mark C. Field, Joel B. Dacks, V. Lila Koumandou

**Affiliations:** ^1^ Genetics Laboratory, Department of Biotechnology Agricultural University of Athens Athens Greece; ^2^ Division of Infectious Diseases, Department of Medicine University of Alberta Edmonton Alberta Canada; ^3^ School of Life Sciences University of Dundee Dundee UK; ^4^ Biology Centre Czech Academy of Sciences České Budějovice Czech Republic; ^5^ Centre for Life's Origin and Evolution, Department of Genetics, Evolution and Environment University College of London London UK; ^6^ Present address: Department of Agricultural, Food and Nutritional Science, Faculty of Agricultural, Life and Environmental Sciences University of Alberta Edmonton Alberta Canada

**Keywords:** comparative genomics, endomembrane system, endoplasmic reticulum, eukaryogenesis, evolution, last eukaryotic common ancestor, phylogeny, reticulons, vesicular traffic

## Abstract

Endomembrane system compartments are significant elements in virtually all eukaryotic cells, supporting functions including protein synthesis, post‐translational modifications and protein/lipid targeting. In terms of membrane area the endoplasmic reticulum (ER) is the largest intracellular organelle, but the origins of proteins defining the organelle and the nature of lineage‐specific modifications remain poorly studied. To understand the evolution of factors mediating ER morphology and function we report a comparative genomics analysis of experimentally characterized ER‐associated proteins involved in maintaining ER structure. We find that reticulons, REEPs, atlastins, Ufe1p, Use1p, Dsl1p, TBC1D20, Yip3p and VAPs are highly conserved, suggesting an origin at least as early as the last eukaryotic common ancestor (LECA), although many of these proteins possess additional non‐ER functions in modern eukaryotes. Secondary losses are common in individual species and in certain lineages, for example lunapark is missing from the Stramenopiles and the Alveolata. Lineage‐specific innovations include protrudin, Caspr1, Arl6IP1, p180, NogoR, kinectin and CLIMP‐63, which are restricted to the Opisthokonta. Hence, much of the machinery required to build and maintain the ER predates the LECA, but alternative strategies for the maintenance and elaboration of ER shape and function are present in modern eukaryotes. Moreover, experimental investigations for ER maintenance factors in diverse eukaryotes are expected to uncover novel mechanisms.

## Graphical abstract

More than 15 proteins have a role in shaping the endoplasmic reticulum (ER) but studies on the evolution of these factors are lacking. Now, a molecular evolutionary analysis indicates that reticulons, REEPs, atlastin, spastin, lunapark, TMEM33, STIM1 and VAPs likely evolved as early as the last eukaryotic common ancestor (LECA). Secondary losses are common, while more recent innovations are evident in the Opisthokonta. Future experimental investigations for these ER maintenance factors in diverse eukaryotes are expected to uncover novel mechanisms.

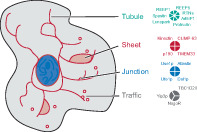

## 1 INTRODUCTION

The eukaryotic endomembrane system mediates export of macromolecules, uptake of molecules and particles from the environment, together with degradation and intracellular transport of proteins, lipids and nutrients.^1^ A central compartment is the endoplasmic reticulum (ER), where nascent membrane and secretory proteins are translocated, folded and transported to the Golgi complex for modification, packaging into vesicles and targeting to the plasma membrane or internal organelles.^2^ By contrast, endocytosed material is packaged into vesicles at the plasma membrane and trafficked to endosomes from where it is either recycled to the plasma membrane or proceeds to late endosomes, multivesicular bodies and the lysosome. Defective cellular components can also be directed to the lysosome for degradation, via autophagy.^3^ Retrograde pathways recycle material from the endosome to the Golgi complex and from the Golgi complex back to the ER.^2^, ^4^

Structural, compositional and functional integrity of the endomembrane system requires the activity of specificity factors and structural proteins directing traffic between compartments; many of these proteins have a clear evolutionary history and arose through paralog expansion.^5^, ^6^, ^7^ Multiple studies have revealed a highly complex last eukaryotic common ancestor (LECA), with compelling evidence that both major organelles and trafficking routes were established before diversification of modern eukaryotic lineages.^8^ A general model for early establishment of ancestral endo‐ and exocytic pathways has emerged.^9^ An archaeal contributor to eukaryogenesis is generally accepted and the Asgard archaea are currently the most likely candidates for this role,^10^ but while potential ancestors of eukaryotic compartment specificity proteins are present in these prokaryotes, there is no evidence for compartmentalization based on mechanisms homologous to eukaryotic systems, and images from the first cultured representative Asgard archaea did not reveal internal membraneous structures.^11^ Overall, the most parsimonious model is that the majority of the endomembrane system arose between an earlier ‘first’ eukaryotic common ancestor (FECA) and LECA, but only general principles of this process are understood.^12^, ^13^

The LECA possessed mitochondria, substantial internal differentiated compartments and a well‐defined nucleus, but the order in which each arose remains unclarified.^8^, ^14^ There are multiple models for the origin of the ER^15^ which are constrained by the presence of the Sec61 translocon, homologous with the SecY bacterial/archaeal export system.^16^, ^17^ In mitochondria‐early models, acquisition of the mitochondrion drives development of the endomembrane system, with the ER as an elaboration of the mitochondrial outer membrane.^18^, ^19^ In mitochondria‐late models, the ER arose by elaboration of either the plasma membrane or the nuclear envelope.^14^ *N*‐Glycosylation, a major ER function, likely originated in archaea, suggesting that quality control and ER‐associated degradation were also present in the LECA.^20^

**FIGURE 1:**
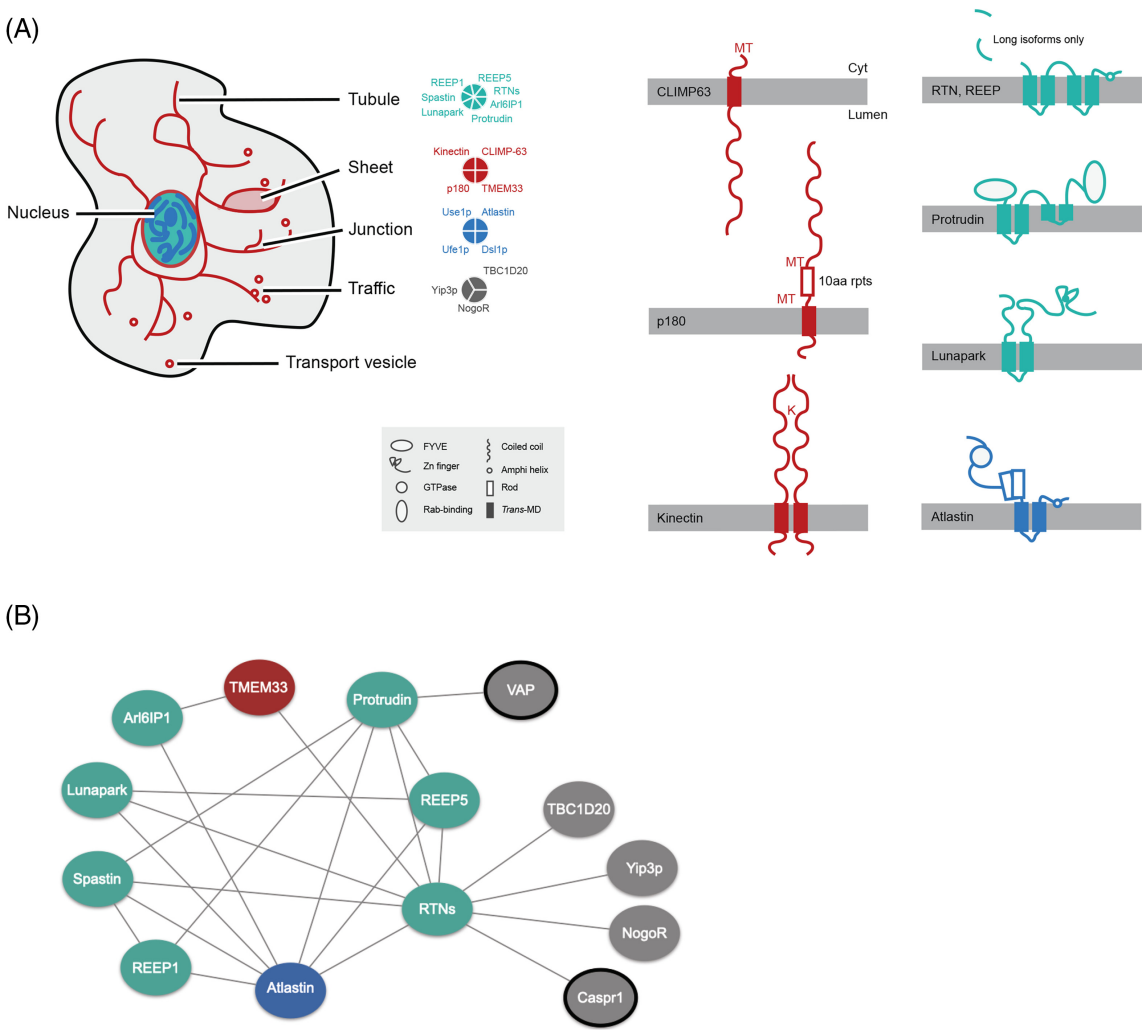
Location, architecture and interactions of ER morphology proteins. Proteins are colour‐coded based on their function in shaping ER tubules (teal), sheets (red), junctions (blue) and other functions in trafficking (grey). A, Schematic of the location and domain architecture of ER morphology proteins. Membrane‐spanning proteins are shown with the cytoplasmic side facing up. Schematically represented domains are shown in the inset. The approximate binding positions of CLIMP‐63 and p180 to microtubules (MT), and of kinectin to kinesin (K) ar also indicated. B, Protein–protein interactions between ER morphology proteins. For further details see Table S1. VAP and Caspr1 are not shown in panel A. Arl6IP1, ADP‐ribosylation factor‐like 6 interacting protein 1; Caspr1, contactin‐associated protein 1; CLIMP‐63, cytoskeleton‐linking membrane protein 63; Dsl1p, depends on SLY1‐20; NogoR, Nogo receptor; p180,180 kDa ribosome receptor; REEP, receptor expression‐enhancing proteins; RTNs, reticulons; TBD1D20, Rab1 GAP TBC‐domain family 20; TMEM33, *trans*‐membrane protein 33; Ufe1p, unknown function essential; Use1p, unconventional SNARE in the ER; VAP, VAMP‐associated protein; Yip3p, Ypt‐interacting protein 3.

Studies of the evolution of factors shaping the ER are lacking and many of the proteins involved have no obvious relationship to general endomembrane specificity factors, precluding inclusion into many prior models of organelle origins.^21^ The ER is a network of sheet‐like cisternae and interconnected tubules and in most cells is contiguous with the nuclear envelope (Figure 1A). Tubule formation is mediated by reticulons, REEP5/DP1/Yop1, REEP1, Arl6IP1/ARMER, spastin, lunapark and protrudin.^22^, ^23^, ^24^ Furthermore, Atlastin/Sey1p, Use1p, Ufe1p and Dsl1p play critical roles in tubule homotypic fusion leading to the emergence of ER junctions and branches.^22^, ^23^, ^24^, ^25^, ^26^, ^27^ CLIMP‐63, kinectin, p180, TMEM33, as well as reticulons are thought to regulate the sheet‐like ER conformation.^22^ In mammalian cells ER organization depends on the cytoskeleton: REEP1 interacts directly with microtubules through a C‐terminal cytoplasmic domain,^28^ while STIM1 is concentrated at ER‐tubule tips and mediates tip attachment complex (TAC) functions, a mechanism by which ER tubules extend along microtubules.^29^

Reticulons orthologs are present in mammals, fungi, amoebozoa and plants, exhibit distinct tissue‐specific expression patterns^30^, ^31^ and are involved in diverse functions, including ER network formation, ER‐Golgi trafficking and apoptosis.^32^ As such, reticulons are implicated in various neurodegenerative diseases, including Alzheimer's dementia, amyotrophic lateral sclerosis, multiple sclerosis and hereditary spastic paraplegia (HSP). Reticulons share the eponymous reticulon homology domain (RHD) near the C‐terminus which consists of two short hairpin *trans*‐membrane domains and is important for subcellular localization and protein–protein interactions.^21^, ^30^, ^32^ Reticulons, REEPs and Arl6IP1 are involved in forming high‐curvature tubular polygonal networks through their double hairpin *trans*‐membrane segments (Figure 1A), which can form a wedge conformation.^22^, ^23^, ^33^ Reticulons can act synergistically with REEPs, while oligomerization into immobile higher‐ordered structures is a requirement for proper tubule formation.^29^ Arl6IP1‐regulated ER tubulation is only thought to be characteristic of metazoa.^33^ Spastin, a disease gene associated with HSP, is a microtubule‐severing AAA ATPase,^34^ and the M1 isoform, through a hairpin partially inserted in the ER membrane, participates in ER network formation.^35^ Spastin also interacts with protrudin, atlastin and REEP1.^22^ Protrudin and lunapark are structurally similar proteins with an antagonistic role towards atlastin in ER‐tubule fusion.^36^, ^37^ Atlastins have only been found in metazoa but similar functions are supported in other eukaryotes by Sey1p.^25^, ^38^ Use1p, Ufe1p and Dsl1p are involved in an atlastin/Sey1p‐independent ER fusion pathway in *Saccharomyces cerevisiae*.^27^, ^38^, ^39^ CLIMP‐63, kinectin and p180 each possess coiled‐coil domains (Figure 1A), important for controlling shape and stacking of ER sheets.^40^, ^41^

Many of these proteins have additional interactors (Figure 1B and Table S1) or function in other aspects of intracellular trafficking. For example, reticulons associate with Yip3p/PRA1 (prenylated Rab acceptor), a guanine dissociation factor, and with TBC1D20, a GTPase‐activating protein which modulates Rab1 and Rab2 activity.^32^, ^42^, ^43^ VAP‐A affects the subcellular localization of protrudin,^44^ while Ufe1p, Use1p and Dsl1p are also involved in retrograde vesicular transport.^45^, ^46^ Nogo‐A (RTN4A) is bound by the Nogo receptor (NogoR), a brain‐specific, leucine‐rich‐repeat protein, an interaction sufficient to inhibit neurite outgrowth in the central nervous system.^47^ Nogo‐A also interacts with the cell adhesion molecule Caspr1, important for localizing potassium channels at axonal paranodes^32^, ^48^ and for propagation of action potentials, obviously animal‐specific functions.

The ER is highly extensive, contributing up to ~50% of total membrane in mammalian cells and, for some eukaryogenesis models, ER origin is crucial for understanding endomembrane system evolution.^49^ Using comparative genomics and phylogenetics we reconstruct the evolution of factors shaping the ER together with protein interactors. While we find a highly conserved core, there is also evidence for post‐LECA diversification, indicating ongoing adaptation of the ER.

## 2 RESULTS AND DISCUSSION

**FIGURE 2:**
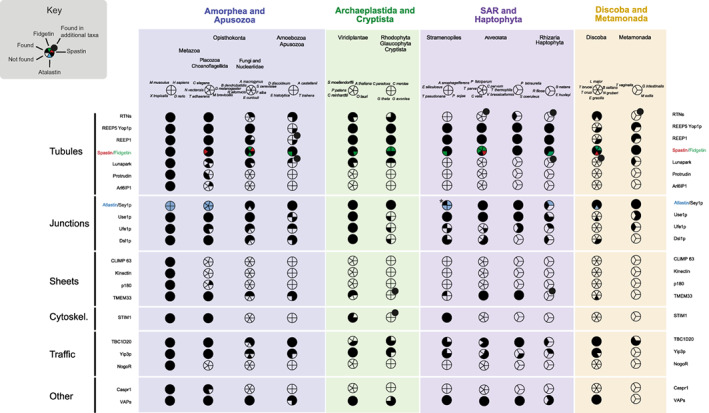
Distribution of ER morphology proteins across eukaryotic lineages. Data are based on BLAST and HMMer results together with alignments and phylogenetic reconstruction. Filled sectors indicate the presence of the protein and empty sectors indicate that the relevant gene was not found. Large taxon groupings are colour coded, and the proteins are grouped based on their function in shaping the morphology of the ER, as in Table 1. Accession numbers are given in Table S3, and complete species names in Table S5. For spastin/fidgetin, the black colour indicates presence of both, pink indicates presence of spastin only, and green indicates presence of fidgetin only (Figure S3). *For Atlastin/Sey1p, blue colour indicates presence of Atlastin, black indicates presence of Sey1p, *Ectocarpus siliculosus* has both (Figure S4). For lineages checked with wider species sampling on the EukProt server (Table S6) a tangential bullet indicates that positive hits were found in other species of that lineage.

**FIGURE 3:**
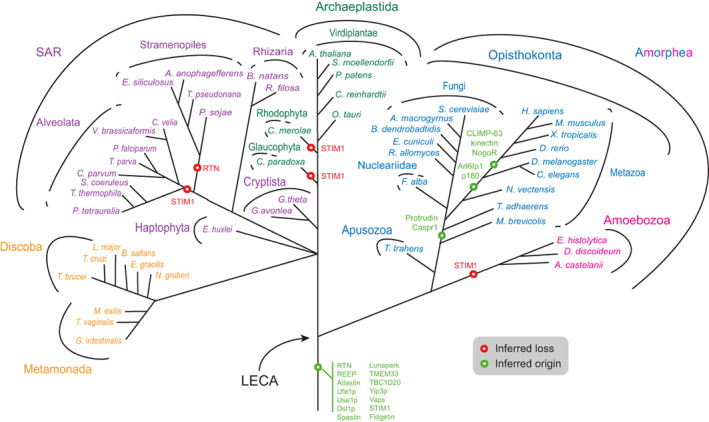
Distribution of ER morphology proteins mapped onto eukaryotic phylogeny. The most likely point of origin of each protein is indicated, based on the results of the present study. The tree omits much detail regarding losses of paralogs from specific taxa and ignores any potential lateral gene transfer. STIM1: most likely origin, but with multiple secondary losses (also see Figure 2). Sey1p, synthetic enhancer of Yop1p; LECA, last eukaryotic common ancestor; STIM1, stromal interaction molecule 1, other abbreviations as in Figure 1.

To reconstruct the evolutionary history of proteins involved in ER morphology, we searched 50 high quality predicted proteomes using protein sequences of ER morphology‐associated proteins from *S. cerevisiae* and *Homo sapiens* as queries; additional criteria, including best reciprocal BLAST, HMMer and retention of domain architecture were also employed (see Section 4). The distribution of these proteins based upon recovered homologs is shown in Figure 2. The major feature to emerge is exceptional widespread conservation, indicating an ancient origin for much of the machinery supporting ER structure and function prior to the LECA (Figure 3 and Table 1). For clarity, we consider proteins below according to their described functions.

**TABLE 1:** List of proteins included in this study, categorized based on their functions relevant to Endoplasmic reticulum (ER) structure and/or other functions, where relevant.

Functions		Proteins	Origin
ER structure	Tubules	Reticulons	Ancient
		REEP5/Yop1p	Ancient
		REEP1	Unclear
		Spastin	Ancient
		Lunapark	Ancient
		Protrudin	Holozoa
		Arl6IP1	Metazoa
	ER junctions	Atlastin/Sey1p	Ancient
		Ufe1p‐Use1p‐Dsl1p	Ancient
	Sheets	Reticulons	Ancient
		CLIMP‐63	Metazoa
		Kinectin	Metazoa
		p180	Metazoa
		TMEM33	Ancient
ER‐microtubules		STIM1	Ancient
		Spastin	Ancient
		CLIMP‐63	Metazoa
		REEP1	Unclear
Endomembrane trafficking		Reticulons	Ancient
		Spastin	Ancient
		Ufe1p‐Use1p‐Dsl1p	Ancient
		TBC1D20	Ancient
		Yip3p	Ancient
		VAPs	Ancient
Other functions		NogoR	Metazoa
		Caspr1	Holozoa

*Note*: Some proteins (e.g. reticulons) have multiple functions and are listed more than once. In the last column, the most probable point of origin of each is indicated, based on the results of the present study.

### 2.1 ER tubules

**FIGURE 4:**
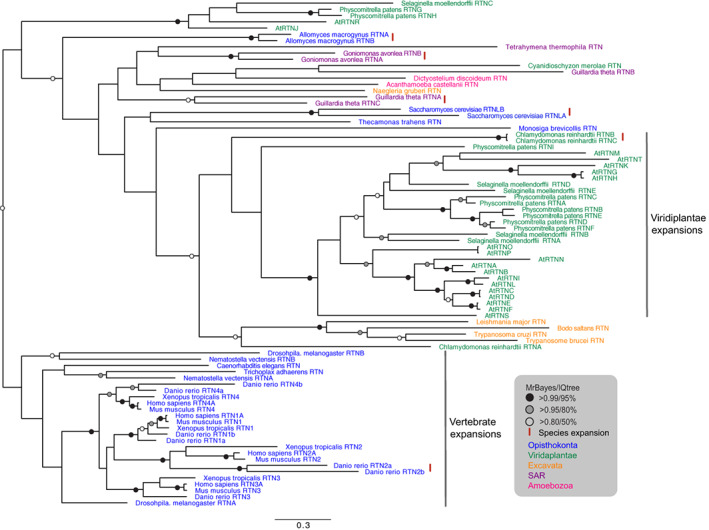
Phylogenetic reconstruction of the reticulons. The tree shown is based on MrBayes. Well‐supported nodes in both the MrBayes and Maximum Likelihood (IQ) analyses are highlighted. The two major expansions in vertebrates and plants are also highlighted. The red lines ‘|’ indicate species‐specific duplications. Species names are coloured as in Figure 3. At, *Arabidopsis thaliana*.

Reticulons and REEP5/Yop1p act synergistically in formation of ER tubules and are highly conserved in multiple lineages (Figures 2 and 3) and likely originated in the LECA.^50^ For reticulons, independent gene duplications occurred in multiple lineages, leading to the emergence of two reticulon proteins in fungi (*S. cerevisiae*, *Allomyces macrogynus*), multiple proteins in Cryptophyta (three in *G. theta* and two in *G. avonlea*) and many in plants (Figure 4 and Table S2). The duplications that led to emergence of four reticulon paralogs in *H. sapiens* must have taken place in the common ancestor of the vertebrates (Figure 4).

The REEP family has six paralogs in *H. sapiens*, which are highly similar. Therefore, discriminating between REEP1 and REEP5 from BLAST and HMMer searches was only possible in vertebrates (*Mus musculus*, *Danio rerio* and *Xenopus tropicalis*). Phylogenetic reconstruction suggests an early duplication in the Opisthokonta leading to two clades, one with vertebrate REEPs 1–4, and one with REEPs 5–6 (Figure S1). Except for *S. cerevisiae*, *Rozella allomycis* and *Thecamonas trahens*, all other opisthokonts have at least two REEP paralogs and all metazoa have a representative in each of the two clades. Furthermore, lineage‐specific duplications are seen for *D. rerio*, *Drosophila melanogaster* and *A. macrogynus*. Expansions of the reticulon and REEP protein families (as well as the VAPs, see below) are particularly common within the Viridiplantae (Table S2). This is consistent with many examples of expansions in the endomembrane system protein cohort in higher plants^51^ and may be related to the frequent whole genome duplications known to have occurred in this lineage.^52^, ^53^, ^54^ In addition, these expanded protein families may be associated with tissue‐specific functions.^55^, ^56^ In metazoa, there is also evidence for tissue or developmental‐linked expression for reticulon paralogs and their isoforms, as well as for REEPs^30^, ^31^, ^57^, ^58^, ^59^; it is thus probable that expansions of these protein families in other organisms resulted in differentiated functions.

Lunapark is conserved in metazoa, plants, *Dictyostelium discoideum*, *Cyanidioschyzon merolae*, *Cyanophora paradoxa* and *Trichomonas vaginalis*. Notably, lunapark was not found in the Stramenopiles and Alveolata. Lunapark antagonizes atlastin in the fusion of ER tubules and stabilizes nascent three‐way junctions.^37^, ^60^ Furthermore, a theoretical model has been proposed explaining ER morphologies and remodelling based on only two types of curvature‐stabilizing proteins that generate straight or concave sheet edges, exemplified by the reticulons and lunapark, respectively.^61^ Therefore, in organisms lacking lunapark, either its function is performed by a non‐homologous protein or some other mechanism exists to control the dynamics between ER tubules and sheets.

Protrudin and Arl6IP1 are also involved in formation of tubular ER and as both are only present in opisthokonts are thus lineage‐specific. Protrudin emerged in the placozoa to regulate ER density and the ratio between tubules and sheets. Important for protrudin localization is interaction with VAP‐A^62^; VAP (vesicle‐associated membrane protein‐associated protein) also contributes to tethering between the ER and the plasma membrane. A second isoform, VAP‐B, is found in mammals, mutants of which give rise to amyotrophic lateral sclerosis and induce ER restructuring.^44^ VAP paralogs have many roles in intracellular trafficking and are localized to the Golgi, ER‐Golgi intermediate compartment, tight junctions, neuromuscular junctions, recycling endosomes, and the plasma membrane.^62^ Unsurprisingly, therefore, VAPs are widely conserved (Figures 2 and 3), but while multiple lineage‐specific expansions are present, VAP‐A and VAP‐B paralogs likely arose in vertebrates (Figure S2).

Arl6IP1 is present only in some metazoa, in concordance with previous studies, suggesting that Arl6IP1 participates in formation of the ER tubules only in this lineage.^33^ Arl6IP1 recruits the inositol 5‐phosphatase INPP5K (SKIP) to the ER and specifically to newly formed ER tubules that grow along microtubule tracks.^63^ Therefore, this mechanism for recruitment of INPP5K is apparently specific to some metazoa and, significantly, absence from *Caenorhabditis elegans* indicates a distinct phosphoinositide signalling platform. Interestingly, Arl6IP1 also interacts with atlastin‐1, is an antiapoptotic protein, is the conophylline receptor as well as the genetic determinant for HSP and pain insensitivity,^33^, ^64^, ^65^ which may well be linked with inositol phosphate signalling.

Spastin belongs to the diverse AAA‐ATPase superfamily which share a common ATPase domain and spastin could not always be unequivocally determined from initial searches. In many cases the top BLAST hits were annotated as ‘fidgetin’, which is a spastin paralog. We carried out phylogenetic analysis for all spastin and fidgetin sequences in our species of interest, which showed that multiple species across all eukaryotic lineages have both spastin and fidgetin, suggesting an early origin for both (Figure S3). *Nematostella vectensis*, *S. cerevisiae*, *Vitrella brassicaformis* and *Euglena gracilis*, only have spastins, but a number of species outside the metazoa only have fidgetin, including *Encephalitozoon cuniculi*, *A. macrogynus*, *Entamoeba histolytica*, *Ostreococcus tauri*, *Cyanophora paradoxa*, *C. merolae*, *Thalassiosira pseudonana*, *Plasmodium falciparum*, *Theileria parva*, *Cryptosporidium parvum*, *Emiliania huxleyi*, *Bodo saltans* and *Leishmania major*; the rather patchy representation likely indicates multiple secondary losses of one or other paralog. However, none of the species studied has lost both paralogs, suggesting an important function. Because both proteins function as microtubule‐severing enzymes,^66^ and have only been marginally studied outside metazoa,^67^ experimental work is needed to confirm the roles of spastin and fidgetin across the eukaryotes, and their potential contributions towards ER topology.

### 2.2 ER junctions

Homotypic membrane fusion of neighbouring ER tubules is mediated by atlastin and Sey1p, which have very similar functions.^68^ Although the proteins have overall low sequence similarity, both belong to the dynamin family and possess a cytosolic N‐terminal GTPase domain, followed by a helical bundle domain, which is significantly longer in Sey1p, two closely spaced *trans*‐membrane segments and a cytosolic C‐terminal tail, which includes an amphipathic helix.^27^ Our analysis indicates that eukaryotes possess either an atlastin or a Sey1p ortholog, except for *Ectocarpus siliculosus* for which an ortholog for both can be identified (Table S3 and Figure S4). Atlastins are found in metazoa, stramenopiles, *Bigelowiella natans* and *Euglena gracilis*, whereas Sey1p is present in all other organisms. Previous analyses^25^, ^38^ suggested a model in which ancestral Sey1p mediated homotypic membrane fusion in the LECA with atlastin emerging in the metazoa, but the fact that we see multiple examples of atlastin outside metazoa may instead indicate that both proteins were present in LECA, followed by multiple losses. The longer helical bundle of Sey1p‐like proteins is important for dimerization^69^; however, atlastins also dimerize^27^, ^68^ so the longer helical bundle domain may also have another role, e.g. in spacing of ER junctions. Further analysis, including wider species sampling and other homologous proteins may help clarify the origin of atlastin and Sey1p, as well as differences in their function.

Atlastin is likely the sole mediator of ER fusion in metazoa, as is the Sey1p‐homolog RHD3 in plants. However, an alternative fusion pathway in *S. cerevisiae* is mediated by the ER SNAREs Ufe1p and Use1p in Sey1p‐mutant cells, which also requires the tethering protein Dsl1p,^27^, ^38^, ^39^ although it is not known if this occurs in parallel with the Sey1p‐mediated mechanism in wild type cells, or only compensates Sey1p mutants. Ufe1p, Use1p, and Dsl1p are widely distributed in eukaryotes (Figures 2 and 3, Table S4,^70^, ^71^), which is likely explained by their central role in retrograde vesicular transport.

### 2.3 ER sheets

Reticulons, CLIMP‐63, kinectin, p180 and TMEM33 are involved in formation of ER sheets. Reticulons locate to the edges of ER sheets generating high‐membrane curvature^22^ and are highly conserved. CLIMP‐63, kinectin and p180 are non‐essential for ER sheet formation^40^ and are present only in some metazoa (Figures 2 and 3). Our results indicate that CLIMP‐63 and kinectin originated before the evolution of *D. rerio* and p180 before *D. melanogaster*. In contrast, orthologs of TMEM33 are found in at least some species of most lineages: opisthokonts, fungi, apusozoa, ameobozoa, plants, glaucophyta, rhodophyta, stramenopiles, alveolata, rhizaria and discoba (Figures 2 and 3). These results indicate an ancient role for reticulons outside metazoa in the formation of the ER sheets, most likely in stabilizing the edges,^40^ and for TMEM33, which binds to reticulon homology domain‐containing proteins and regulates their membrane‐shaping activity.^22^ CLIMP‐63, kinectin and p180 are coiled‐coil domain proteins promoting sheet formation, further antagonizing the curvature‐promoting action of reticulons; these proteins also associate with polysomes, characteristic of rough ER, and optimize the size of the luminal space of ER sheets,^40^ while recent evidence suggests preferential interactions with different microtubule populations.^72^ One more metazoa‐specific ER sheet‐promoting protein, TMEM170A, was identified recently.^73^

### 2.4 ER‐cytoskeleton interactions

ER tubules use at least two mechanisms to extend along microtubules, the TAC and ER‐sliding dynamics. TAC functions are mediated by the integral ER membrane protein STIM1, which concentrates at the tip of ER tubules, and the microtubule end‐binding protein 1 (EB1) which localizes to the tip of dynamic microtubules. STIM1 and EB1 interact with each other directly, allowing ER tubules to elongate or contract.^29^, ^74^ STIM1 is conserved across metazoa, viridiplantae and stramenopiles (Figure 2). It is probable that STIM1 arose early in eukaryotic evolution but has been lost frequently, indicating that other proteins are needed for ER‐cytoskeleton interactions. In mammalian cells, spastin, CLIMP‐63, p180 and REEP1 all bind microtubules,^28^, ^40^ but it is unknown if they function similarly to STIM1. Spastin and REEP1 interact with atlastin, an association that may aid ER tubules and cytoskeleton microtubules to form an organized network.^28^

### 2.5 Lineage‐specific ER evolution

Multiple duplications were observed for many of the proteins considered here, including reticulons, REEP5/Yop1p, atlastin/Sey1p, Yip3p and VAPs, indicating species‐specific and lineage‐specific innovation. Furthermore, secondary losses are common in individual species, and even in certain lineages, for example the reticulons have most likely been lost from the stramenopiles; lunapark from the stramenopiles and alveolata; TMEM33 from the cryptista, haptophyta and metamonada, and STIM from the alveolata, excavata, glaucophyta, haptophyta, rhizaria and rhodophyta. To further check whole‐lineage losses apparent in Figure 2, extra analyses were done using wider species sampling, the results of which are shown in Table S6. For example, REEP has been lost in the three amoebozoa species initially examined (*E. histolytica*, *D. discoideum*, *Acanthamoeba castellanii*) but further analysis showed that REEP homologs are present in certain other species of this lineage (*Dracoamoeba jomungandri*, *Filamoeba sp_ATCC50430*, *Vermamoeba vermiformis*, *Arcella intermedia*, *Amoeba proteus*).

If protein distribution is considered by organism, a number of interesting features emerge. The most dramatic is absence of several proteins involved in ER formation from a number of lineages. More specifically, atlastin/Sey1p, spastin and Yip3 were the only proteins recovered in *E. histolytica*, and atlastin/Sey1p, spastin, Use1p and TBC1D20 the only proteins recovered in *Monocercomonoides exilis* (Figure 2); notably, the GTPase substrates of TBC1D20, Rab1 and Rab2, are also conserved in *M. exilis* giving confidence for this result.^75^ The apparent absence of ER shaping factors in certain lineages could be attributed to increased sequence divergence or genome data limitations, but multiple absences make this unlikely and these observations potentially indicate that mechanisms for ER formation are highly simplified or mediated by novel factors in those organisms. Indeed, the non‐classical structure of the ER in *E. histolytica* ^76^, ^77^ and *M. exilis* ^75^, ^78^ could be attributed to the lack of reticulons and REEPs. A final significant feature is the presence of multiple paralogs (three REEPs, two atlastin/Sey1p proteins, three spastins/fidgetins, three Use1 proteins, three TMEM33, two TBC1D20 and seven VAPs) in *Paramecium tetraurelia* (Table S2). *P. tetraurelia* has nearly 40 000 genes, most of which arose through at least three successive whole‐genome duplications, likely explaining these features.^79^ The different paralogs may also play a role in remodelling the ER during different life stages in *Paramecium*.^80^ [Correction added on 30 August 2022, after first online publication: The text “*Mammuthus exilis*” in the second paragraph of page 8 has been corrected to “*M. exilis*”.]

Animal‐specific interactors of reticulons include NogoR and Caspr1. NogoR, the receptor for RTN4A (NogoA), is localized at the plasma membrane of neurons, and binding of RTN4A to NogoR can lead to inhibition of neuronal growth.^32^ This receptor appears to be present only in vertebrates: *H. sapiens*, *M. musculus*, *X. tropicalis*, *D. rerio* (Figure 2). This narrow distribution can be explained by the presence of a highly developed nervous system in these organisms. Caspr1 (contactin‐associated protein) belongs to a family of transmembrane proteins participating in forming and stabilizing myelinated axons^81^ and interacts with RTN4A to mediate localization of potassium channels in axonal paranodes.^32^ Metazoa and placozoa have at least one member of the Caspr protein family (Figures 2 and 3, Table S2), although for *D. melanogaster*, *C. elegans* and *Trichoplax adhaerens* it remains to be determined if these are true Caspr1 orthologs (Table S3). Notably, *T. adhaerens* lacks a typical nervous system with axons, synapses or muscles,^82^ so a Caspr protein in this organism would likely have a different function. Interestingly, Caspr is also conserved in gastropods (results not shown), which also have a highly developed nervous system, but which evolved along distinct lines and independently from the metazoan system.

## 3 CONCLUSIONS

The endomembrane system comprises multiple organelles providing important functions specific to eukaryotic cells. Significantly, many of the ER proteins studied here are widely distributed across eukaryotes, pointing to an origin predating the LECA and diversification of eukaryotic supergroups. Apart from being fully consistent with a highly complex endomembrane system in the LECA, these observations unite the ER with other compartments in terms of an ancient origin.^6^, ^83^, ^84^ Reticulons/REEPs, spastin/fidgetin, atlastin/Sey1p, TMEM33 and STIM delineate a minimum set of ancient proteins for shaping major ER features, namely tubules, junctions, sheets and cytoskeletal interactions. Added to this are further elaborations in the opisthokonta (Protrudin, Arl6IP1, CLIMP‐63, kinectin, p180, NogoR and Caspr1), and involvement of factors which have functions outside ER formation (Use1p, Ufe1p, Dsl1p, Vaps, TBC1D20 and Yip3). Secondary losses and lineage‐specifc duplications are common, with some evidence from metazoa and higher plants for differentiated functions between paralogs.

Several central components of the ER have clear antecedents in the Archaea. Use of dolichol‐pyrophosphate as a lipid‐linked oligosaccharide donor in Archaea is in common with eukaryotes as opposed to dolichol phosphate as used by bacteria,^85^ while the Archaeal universal signal recognition particle protein SRP54 is more closely related to eukaryotes than bacteria, and SRP19 is present in Archaea but not bacteria.^86^ However, the ER protein translocase in Archaea is simpler than eukaryotes, and similarly some components of the ER quality control and glycosylation apparatus must post‐date FECA. Evolution of the machinery required to build and maintain an internal fenestrated network of membranes together with protein folding and quality control mechanisms was therefore clearly complete by the time of LECA. Functional studies of the factors shaping the ER in organisms outside the opisthokonta would greatly enhance our understanding of the flexibility of this organelle across the eukaryotes.

## 4 METHODS

### 4.1 Databases

Data were collected from 50 species with high quality genome databases and selected to provide a wide sampling of the eukaryotic super‐groups,^87^ including multiple representative taxa in each group. The choice of species was such as to facilitate detection of species‐specific secondary losses versus absence from the group, to minimize detection failure because of species‐specific sequence divergence and so that failure to retrieve a candidate ortholog could be ascribed to true absence or extreme divergence, but not database incompleteness. Details of databases used are given in Table S5. Predicted proteomes for most species were downloaded from the respective databases for local analysis.

### 4.2 Taxonomic homology survey

Initial queries used *H. sapiens* and *S. cerevisiae* predicted proteins (Tables S2 and S3). Forward BLAST^88^ searches were run using default settings and an *e*‐value cut‐off of 0.05. A relatively high *e*‐value was selected to reduce the number of sequences falsely excluded because of sequence divergence. All recovered sequences were subjected to reverse BLAST against the original genome (i.e. *H. sapiens* or *S. cerevisiae*) and, in some cases, against the NCBI non‐redundant database for confirmation of orthology. For yeast queries, reverse BLAST searches were run manually; for human protein queries, reverse BLAST searches were run automatically (with an *e*‐value cut‐off of 0.05) and further inspected manually. A candidate ortholog was considered if reverse BLAST recovered the original query or annotated orthologs from other species, within the top five hits. Additionally, both for initial candidate identification and for validation by reverse BLAST, rather than relying solely on *e*‐values, sequences were analysed by alignment and parsed through the NCBI conserved domain database for the presence of significant sequence similarity throughout the protein length, conservation of overall protein length and domain architecture. In cases where the initial queries failed to recover a candidate ortholog, the following three strategies were used: Forward BLAST searches were repeated using query sequences (annotated, or retrieved in our analysis) from a taxon more closely related to the target genome (e.g. an *Arabidopdis* protein used as the query against *Chlamydomonas*), HMMer v3.1b1 (hmmer.org) was used with a template composed of the entire set of recovered proteins for a given query and with a cut‐off significance parameter of 0.05, or yeast and/or human protein sequences were used as queries for tBLASTn against genomic contigs. Default tBLASTn settings were used, again with an *e*‐value cut‐off of 0.05. Results from these searches were evaluated for *e*‐value, predicted protein length, conserved domains, and subjected to reverse BLAST against the original query genome. Furthermore, returned candidate sequences were aligned and subject to phylogenetic analysis to confirm both extensive sequence homology and monophyly. Failure to identify a significant hit with all these methods resulted in assignment of ‘not found’. Detailed results from all searches are shown in Table S3. To further check whole‐lineage losses apparent in Figure 2, as well as certain positive outliers (e.g. the *Tetrahymena thermophila* reticulon and the *D. discoideum* lunapark) extra BLAST searches were done using wider species sampling, the results of which are shown in Table S6. For these searches, all available species in the TCS database of the EukProt server^89^, ^90^ (http://evocellbio.com/eukprot/) for each linage of interest were examined by BLAST, using as queries *H. sapiens* and *S. cerevisiae* predicted proteins with an *e*‐value cut‐off of 0.05, and all recovered sequences were subjected to reverse BLAST (with an *e*‐value cut‐off of 0.05) on the NCBI server against the *Homo sapiens*, *S. cerevisiae*, and the general RefSeq database for confirmation of orthology. Any novel hit identified by this method was also used as a query against the rest of the species of the lineage. Specifically, we used the EukProt server to search for (a) reticulons in the Alveolata, Metamonada, Rhizaria and Stramenopiles, (b) REEP in the Amoebozoa, (c) Lunapark in SAR and Discoba, (d) TMEM33 in Cryptista, Glaucophyta, Haptophyta, Metamonada, Rhizaria and Rhodophyta, and (e) STIM in the Alveolata, Amoebozoa, Cryptista, Excavata (Discoba and Metamonada), Glaucophyta, Haptophyta, Rhizaria and Rhodophyta.

### 4.3 Alignments and phylogenetic reconstruction

Alignments (available in the Appendix S1) were created using MUSCLE^91^ and masked to retain only unambiguously homologous regions. Phylogenetic analysis was performed by two separate methods. To obtain the Bayesian tree topology and posterior probability values, MrBayes version 3.1.2 was used,^92^ with the LG model of sequence evolution^93^ and a gamma distribution of four categories of rate. Analyses were run with four chains for 2 x 10^7^ generations, removing all trees before a plateau established by graphical estimation and checked for convergence. All analyses had an average standard deviation of split frequencies less than 0.01 (indicating convergence), with the exception of the pan‐eukaryotic REEP analysis (0.027) and VAP analysis (0.014). Maximum‐likelihood (ML) analysis was performed using IQ‐TREE v.1.6.10^94^ on the CIPRES Science Gateway server.^95^ IQ‐TREE was run with ultrafast bootstrap approximation (UFBoot) to assess branch support.^96^ Model testing was performed using the built‐in ModelFinder program with the best model selected according to the BIC criterion, and 1000 pseudoreplicates were obtained until tree convergence reached the default convergence coefficient.^97^ Trees were visualized in FigTree. Nodes with greater than 0.95 posterior probability and 80% bootstrap support were considered robust, and nodes with over 0.80 posterior probability and 50% bootstrap support are highlighted.

5

### PEER REVIEW

The peer review history for this article is available at https://publons.com/publon/10.1111/tra.12863.

## Supporting information


**Appendix S1** Supporting information.

**Figure S1** Phylogenetic reconstruction of the REEP family in opisthokonts. The tree shown is based on MrBayes. Well‐supported nodes in both the MrBayes and Maximum Likelihood (IQ) analyses are highlighted. The REEP 1–4 and REEP 5–6 clades in vertebartes are also highlighted. The red parentheses ‘)’ indicate species‐specific duplications.

**Figure S2** Phylogenetic reconstruction of the VAPs. The tree shown is based on MrBayes. Well‐supported nodes in both the MrBayes and Maximum Likelihood (IQ) analyses are highlighted. The red parentheses ‘)’ indicate species‐specific duplications or expansions. The duplication leading to mammalian VAP‐A and VAP‐B likely occurred in the vertebrates. Species names are coloured as in Figure 3. At: *Arabidopsis thaliana*

**Figure S3** Phylogenetic reconstruction of spastin and fidgetin. The tree shown is based on MrBayes. Well‐supported nodes in both the MrBayes and Maximum Likelihood (IQ) analyses are highlighted. The tree separates the fidgetin (top) and spastin (bottom) clades. The red parentheses ‘)’ indicate species‐specific duplications. Species names are coloured as in Figure 3.

**Figure S4** Phylogenetic reconstruction of atlastin and Sey1p. The tree shown is based on MrBayes. Well‐supported nodes in both the MrBayes and Maximum Likelihood (IQ) analyses are highlighted. The tree separates the atlastin (top) and Sey1p (bottom) clades; the inset on the left shows the same tree in star format to highlight the clear separation of the two clades (species name abbreviations are as in Table S5). The red parentheses ‘)’ indicate species‐specific duplications. Species names are coloured as in Figure 3.

**Table S1** Protein–protein interactions between the proteins included in this study, based on the literature. Summarized in Figure 1B.

**Table S2** Summary of the comparative genomics results. Human or yeast proteins were used as queries against 50 genomes, representing the full diversity of eukaryotes (see Figure 3 and taxonomic information in the last column). The length and conserved domains of each query protein are given in the top 10 lines. Grey cells with a ‘−’ sign indicate ‘absence’ as no significant hit was found in a genome for the corresponding protein. Green cells with a ‘+’ sign indicate ‘presence’ based on forward and reverse BLAST hits with significant *e*‐value (see Table S3); a number in such cells indicates that >1 ortholog was found. Blue cells with a ‘+’ sign indicate possible ‘paralogs’ based on forward BLAST hits with significant *e*‐value (see Table S3) but where the best reverse BLAST hit was to a different member of the protein family; a number in such cells indicates that >1 homolog was found.

**Table S3** Detailed results of the comparative genomics analysis. For each result, the accession number of the corresponding protein in each genome is given, as well as the protein length, and the *e*‐value of the forward BLAST or HMMer analysis. In cases of multiple isoforms, only the accession number of the longest isoform is given. Grey cells indicate ‘absence’ as no significant hit was found in a genome for the corresponding protein (includes notes on ‘neighbour‐BLAST’ results). Coloured cells indicate ‘presence’ based on forward and reverse BLAST or HMMer hits with significant *e*‐value. Light green: results from BLAST (*e*‐values from HMMer in parentheses), dark green: results from HMMer, blue: results from ‘neighbour‐BLAST’ (*e*‐values from HMMer in parentheses), pink: results from tBLASTn against contigs (mostly retrieves non‐annotated proteins, so the start and end positions of the BLAST hit on the corresponding contig is given). Notes in red indicate discrepancies in protein size between the query and the hit, hinting at possible mis‐annotations or gene fusions/fissions.

**Table S4** A comparison of the results of this study with previous analyses in the literature, for the proteins Use1p and Dsl1p. Previous comparative genomic analysis has been conducted for 2 proteins used in the present study: Use1p (Vankatesh et al., 2017) and Dsl1p (Klinger et al., 2013). Grey cells with a ‘−’ sign indicate ‘absence’ of a protein ortholog. Green cells with a ‘+’ sign indicate ‘presence’ of a protein ortholog; a number in such cells indicates that >1 ortholog was found. Dark cells indicate that the organism was not included in the corresponding study.
**Table S5** List of organisms included in this study. For each species, an abbreviation is given, which was used for the alignments and phylogenetic analyses. The genome source for each organism used for BLAST and Hmmer searches are also indicated.

**Table S6** Detailed results of the comparative genomics analysis for lineages checked with wider species sampling on the EkProt server.
