# Supplementary figures and images for: Evolution of factors shaping the endoplasmic reticulum
Source: Traffic. 2022 Aug 17;23(9):462–73. doi: 10.1111/tra.12863 (PMC9804665; doi:10.1111/tra.12863)

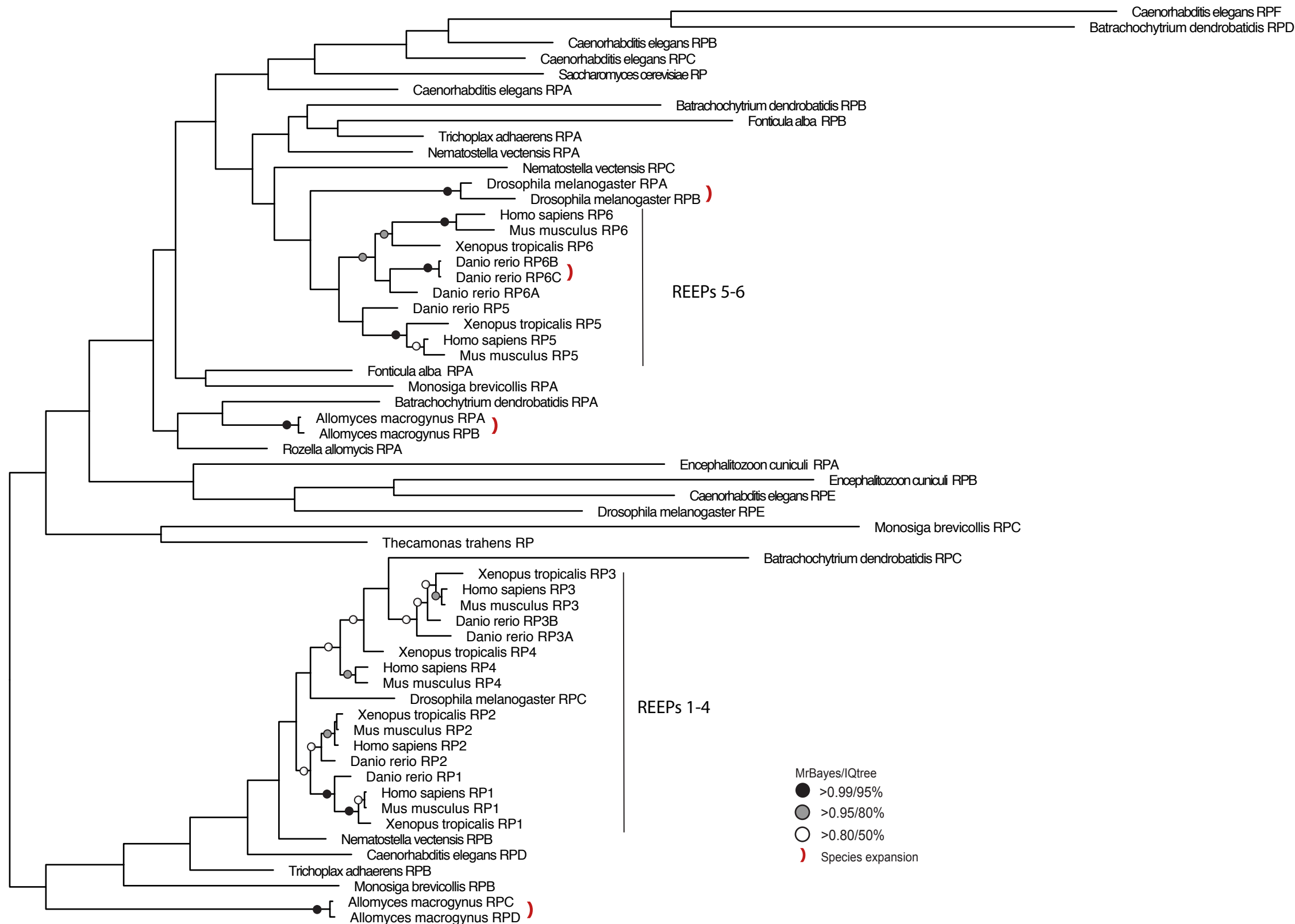

Supplement: Supplementary file 2 — Figure S1 Phylogenetic reconstruction of the REEP family in opisthokonts. The tree shown is based on MrBayes. Well‐supported nodes in both the MrBayes and Maximum Likelihood (IQ) analyses are highlighted. The REEP 1–4 and REEP 5–6 clades in vertebartes are also highlighted. The red parentheses ‘)’ indicate species‐specific duplications. [file TRA-23-462-s009.pdf]

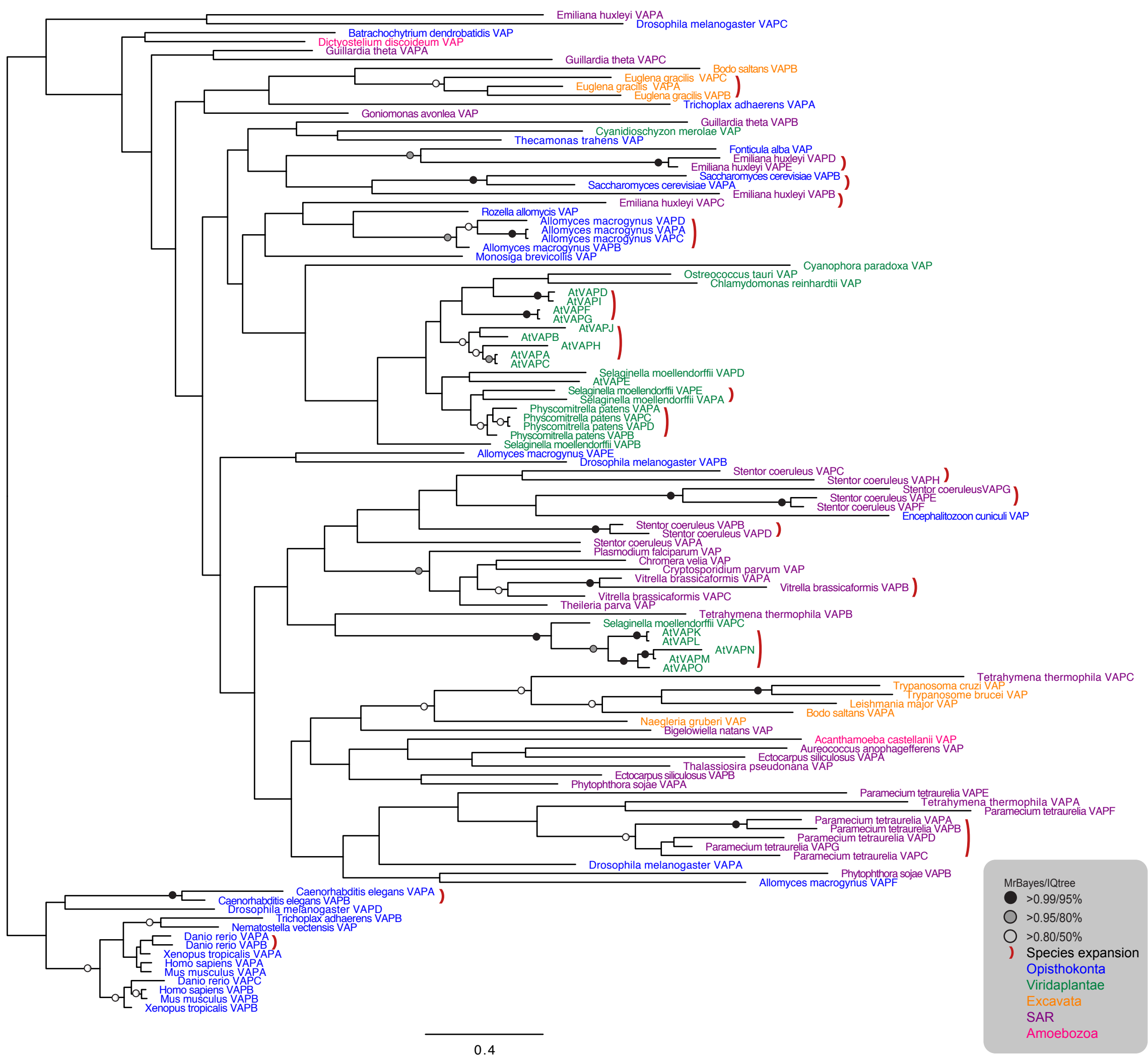

Supplement: Supplementary file 3 — Figure S2 Phylogenetic reconstruction of the VAPs. The tree shown is based on MrBayes. Well‐supported nodes in both the MrBayes and Maximum Likelihood (IQ) analyses are highlighted. The red parentheses ‘)’ indicate species‐specific duplications or expansions. The duplication leading to mammalian VAP‐A and VAP‐B likely occurred in the vertebrates. Species names are coloured as in Figure 3. At: Arabidopsis thaliana [file TRA-23-462-s007.pdf]

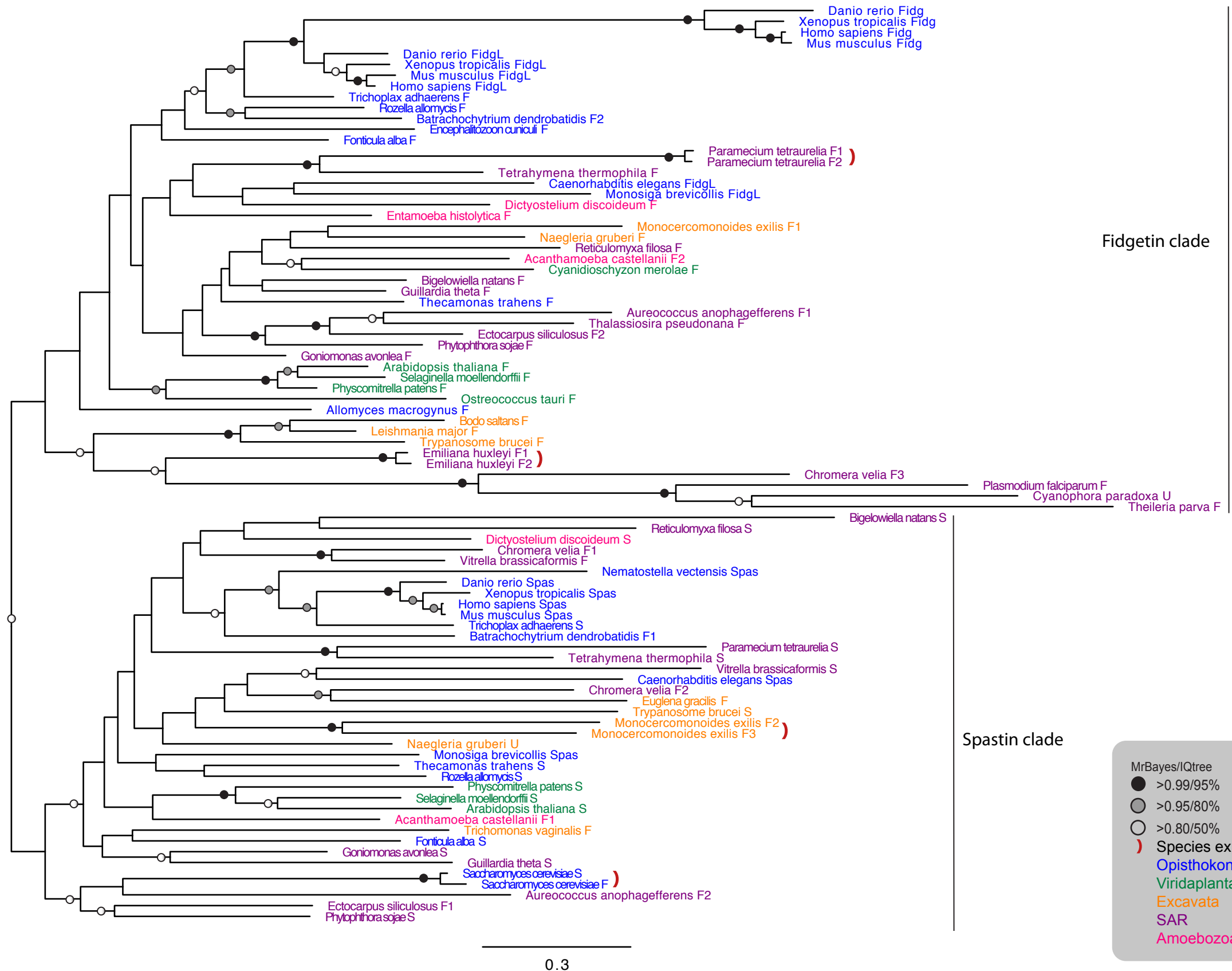

Supplement: Supplementary file 4 — Figure S3 Phylogenetic reconstruction of spastin and fidgetin. The tree shown is based on MrBayes. Well‐supported nodes in both the MrBayes and Maximum Likelihood (IQ) analyses are highlighted. The tree separates the fidgetin (top) and spastin (bottom) clades. The red parentheses ‘)’ indicate species‐specific duplications. Species names are coloured as in Figure 3. [file TRA-23-462-s001.pdf]

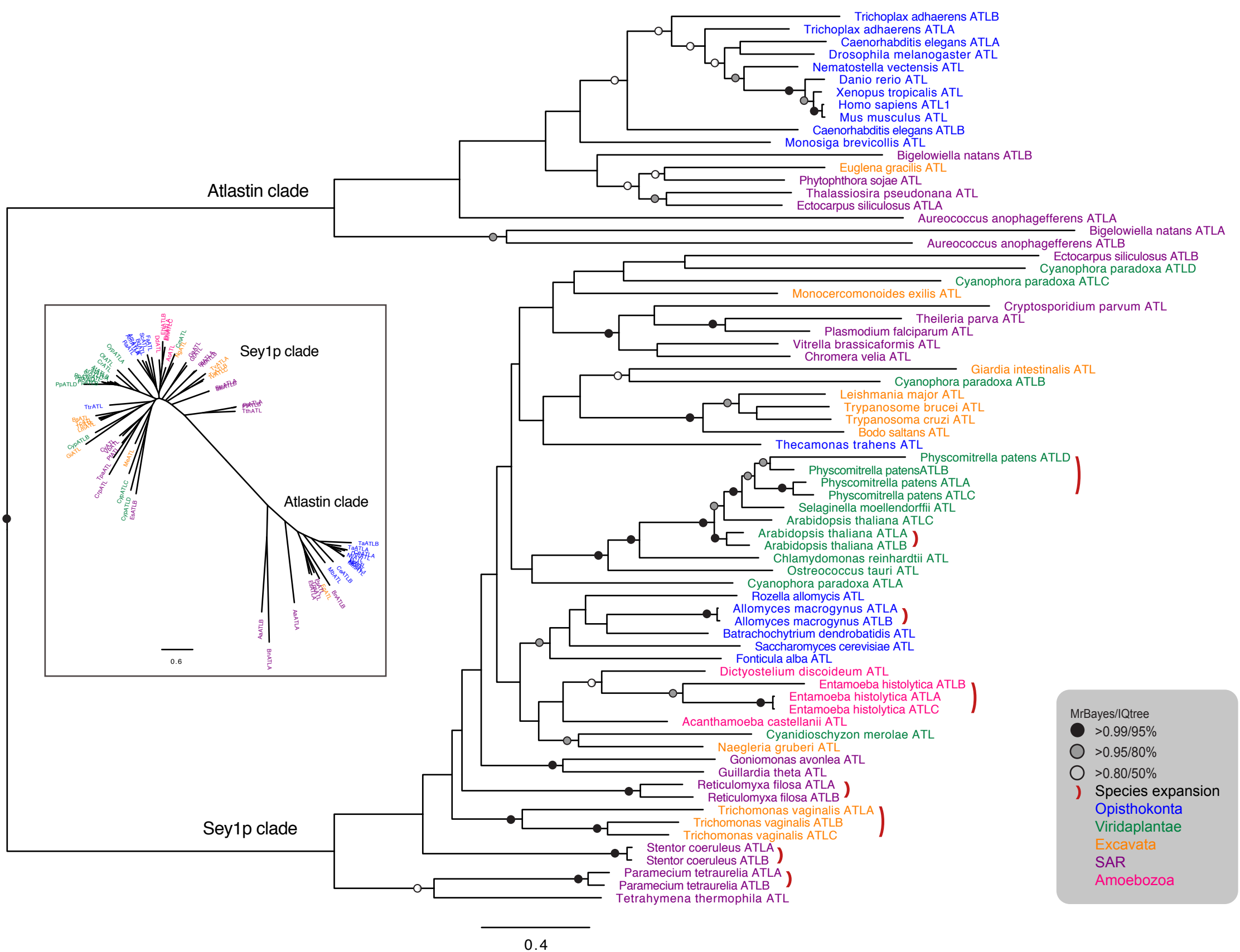

Supplement: Supplementary file 5 — Figure S4 Phylogenetic reconstruction of atlastin and Sey1p. The tree shown is based on MrBayes. Well‐supported nodes in both the MrBayes and Maximum Likelihood (IQ) analyses are highlighted. The tree separates the atlastin (top) and Sey1p (bottom) clades; the inset on the left shows the same tree in star format to highlight the clear separation of the two clades (species name abbreviations are as in Table S5). The red parentheses ‘)’ indicate species‐specific duplications. Species names are coloured as in Figure 3. [file TRA-23-462-s002.pdf]
