# Supplementary material for: Evolution of factors shaping the endoplasmic reticulum
Source: Traffic. 2022 Aug 17;23(9):462–73. doi: 10.1111/tra.12863 (PMC9804665; doi:10.1111/tra.12863)
Supplement: Supplementary file 9 — Table S4 A comparison of the results of this study with previous analyses in the literature, for the proteins Use1p and Dsl1p. Previous comparative genomic analysis has been conducted for 2 proteins used in the present study: Use1p (Vankatesh et al., 2017) and Dsl1p (Klinger et al., 2013). Grey cells with a ‘−’ sign indicate ‘absence’ of a protein ortholog. Green cells with a ‘+’ sign indicate ‘presence’ of a protein ortholog; a number in such cells indicates that >1 ortholog was found. Dark cells indicate that the organism was not included in the corresponding study. Table S5 List of organisms included in this study. For each species, an abbreviation is given, which was used for the alignments and phylogenetic analyses. The genome source for each organism used for BLAST and Hmmer searches are also indicated. [file TRA-23-462-s006.docx]

**Supplementary Figures & Tables:**

**Supplementary Figure S1: Phylogenetic reconstruction of the REEP family in opisthokonts.** The tree shown is based on MrBayes. Well-supported nodes in both the MrBayes and Maximum Likelihood (IQ) analyses are highlighted. The REEP 1-4 and REEP 5-6 clades in vertebartes are also highlighted. The red parentheses “)” indicate species-specific duplications.

**Supplementary Figure S2: Phylogenetic reconstruction of the VAPs.** The tree shown is based on MrBayes. Well-supported nodes in both the MrBayes and Maximum Likelihood (IQ) analyses are highlighted. The red parentheses “)” indicate species-specific duplications or expansions. The duplication leading to mammalian VAP-A and VAP-B likely occurred in the vertebrates. Species names are coloured as in Figure 3. At: Arabidopsis thaliana

**Supplementary Figure S3: Phylogenetic reconstruction of spastin and fidgetin.** The tree shown is based on MrBayes. Well-supported nodes in both the MrBayes and Maximum Likelihood (IQ) analyses are highlighted. The tree separates the fidgetin (top) and spastin (bottom) clades. The red parentheses “)” indicate species-specific duplications. Species species names are coloured as in Figure 3.

**Supplementary Figure S4: Phylogenetic reconstruction of atlastin and Sey1p.** The tree shown is based on MrBayes. Well-supported nodes in both the MrBayes and Maximum Likelihood (IQ) analyses are highlighted. The tree separates the atlastin (top) and Sey1p (bottom) clades; the inset on the left shows the same tree in star format to highlight the clear separation of the two clades (species name abbreviations are as in Table S5). The red parentheses “)” indicate species-specific duplications. Species names are coloured as in Figure 3.

**Supplementary Table S1: Protein-protein interactions between the proteins included in this study, based on the literature.** Summarized in Figure 1B.

**Supplementary Table S2: Summary of the comparative genomics results.** Human or yeast proteins were used as queries against 50 genomes, representing the full diversity of eukaryotes (see Figure 3 and taxonomic information in the last column). The length and conserved domains of each query protein are given in the top 10 lines. Grey cells with a “-“ sign indicate “absence” as no significant hit was found in a genome for the corresponding protein. Green cells with a “+“ sign indicate “presence” based on forward and reverse BLAST hits with significant e-value (see Supplementary Table S3); a number in such cells indicates that >1 ortholog was found. Blue cells with a “+“ sign indicate possible “paralogs” based on forward BLAST hits with significant e-value (see Supplementary Table S3) but where the best reverse BLAST hit was to a different member of the protein family; a number in such cells indicates that >1 homolog was found.

**Supplementary Table S3: Detailed results of the comparative genomics analysis.** For each result, the accession number of the corresponding protein in each genome is given, as well as the protein length, and the e-value of the forward BLAST or HMMer analysis. In cases of multiple isoforms, only the accession number of the longest isoform is given. Grey cells indicate “absence” as no significant hit was found in a genome for the corresponding protein (includes notes on “neighbour-BLAST” results). Coloured cells indicate “presence” based on forward and reverse BLAST or HMMer hits with significant e-value. Light green: results from BLAST (e-values from HMMer in parentheses), dark green: results from HMMer, blue: results from “neighbour-BLAST” (E-values from HMMer in parentheses), pink: results from tBLASTn against contigs (mostly retrieves non-annotated proteins, so the start and end positions of the BLAST hit on the corresponding contig is given). Notes in red indicate discrepancies in protein size between the query and the hit, hinting at possible mis-annotations or gene fusions/fissions.

**Supplementary Table S4: A comparison of the results of this study with previous analyses in the literature, for the proteins Use1p and Dsl1p.** Previous comparative genomic analysis has been conducted for 2 proteins used in the present study: Use1p (Vankatesh et al., 2017) and Dsl1p (Klinger et al., 2013). Grey cells with a “-“ sign indicate “absence” of a protein orthologue. Green cells with a “+“ sign indicate “presence” of a protein orthologue; a number in such cells indicates that >1 orthologue was found. Dark cells indicate that the organism was not included in the corresponding study.

**Supplementary Table S5: List of organisms included in this study.** For each species, an abbreviation is given, which was used for the alignments and phylogenetic analyses. The genome source for each organism used for BLAST and Hmmer searches are also indicated.

Broad: <https://www.broadinstitute.org/>

CryptoDB: <https://cryptodb.org/cryptodb/>

Dictybase: <http://dictybase.org/>

Ensembl: <https://www.ensembl.org/index.html>

Flybase: <https://flybase.org/>

GeneDB: <https://www.genedb.org/>

GiardiaDB: <https://giardiadb.org/giardiadb/>

JCVI: <https://www.jcvi.org/>

JGI: <https://jgi.doe.gov/>

Microsporidia DB: <https://microsporidiadb.org/micro/>

NCBI: <https://www.ncbi.nlm.nih.gov/>

OrcAE: <https://bioinformatics.psb.ugent.be/orcae/overview/Ectsi>

ParameciumDB: <https://paramecium.i2bc.paris-saclay.fr/>

Phytozome: <https://phytozome.jgi.doe.gov/pz/portal.html>

PlamsoDB: <https://plasmodb.org/plasmo/>

Sanger: <https://www.sanger.ac.uk/>

TGD: <http://ciliate.org/index.php/home/welcome>

TrichDB: <https://trichdb.org/trichdb/>

TriTrypDB: <https://tritrypdb.org/tritrypdb/>

| Organism | Abbreviation | BLAST | Hmmer |
| --- | --- | --- | --- |
| *Aureococcus anophagefferens* | Aa | JGI | JGI |
| *Acanthamoeba castellanii* | Ac | Broad | NCBI |
| *Allomyces macrogynus* | Am | Broad | Broad |
| *Arabidopsis thaliana* | At | NCBI | TAIR |
| *Batrachochytrium dendrobatidis* | Bd | JGI | JGI |
| *Bigelowiella natans* | Bn | JGI | JGI |
| *Bodo saltans* | Bs | Genedb (Sanger) | Sanger |
| *Caenorhabditis elegans* | Ce | NCBI | NCBI |
| *Cyanidioschyzon merolae* | Cm | http://czon.jp/ | http://czon.jp/ |
| *Cyanophora paradoxa* | Cyp | cyanophora.rutgers.edu | cyanophora.rutgers.edu |
| *Cryptosporidium parvum* | Crp | CryptoDB | CryptoDB |
| *Chlamydomonas reinhardtii* | Cr | Phytozome | Phytozome |
| *Chromera velia* | Cv | CryptoDB | CryptoDB |
| *Dictyostelium discoideum* | Dd | Dictybase | Dictybase |
| *Drosophila melanogaster* | Dm | FlyBase | FlyBase |
| *Danio rerio* | Dr | NCBI | NCBI |
| *Encephalitozoon cuniculi* | Ec | MicrosporidiaDB | NCBI |
| *Euglena gracilis* (transcriptome) | Eg | Mark Field | Mark Field |
| *Entamoeba histolytica* | Ehi | JCVI | JCVI originally, now on AmoebaDB |
| *Emiliana huxleyi* | Ehu | JGI | JGI |
| *Ectocarpus siliculosus* | Es | OrcAE | JGI |
| *Fonticula alba* | Fa | Broad | Broad |
| *Homo sapiens* | Hs |  |  |
| *Goniomonas avonlea* | Ga |  |  |
| *Giardia intestinalis* | Gi | GiardiaDB | GiardiaDB |
| *Guillardia theta* | Gt | JGI | JGI |
| *Leishmania major* | Lm | TriTrypDB/Sanger | TriTrypDB |
| *Monosiga brevicollis* | Mb | JGI | NCBI |
| *Monocercomonoides exilis* | Me | http://www.protistologie.cz/hampllab/data.html | http://www.protistologie.cz/hampllab/data.html |
| *Mus musculus* | Ms | NCBI | NCBI |
| *Naegleria gruberi* | Ng | JGI | JGI |
| *Nematostella vectensis* | Nv | JGI | NCBI |
| *Ostreococcus tauri* | Ot | JGI | JGI |
| *Plasmodium falciparum* | Pf | PlasmoDB | PlasmoDB |
| *Physcomitrella patens* | Pp | JGI | Phytozome |
| *Phytophthora sojae* | Ps | JGI- v1.1 | JGI- v3.0 |
| *Paramecium tetraurelia* | Pt | ParameciumDB | TGD |
| *Rozella allomycis* | Ra | JGI | NCBI |
| *Reticulomyxa filosa* | Rf | NCBI | Ensembl |
| *Saccharomyces cerevisiae* | Sc |  |  |
| *Selaginella moellendorffii* | Sm | Phytozome | JGI |
| *Stentor coeruleus* | Stc |  |  |
| *Trichoplax adhaerens* | Ta | JGI | JGI |
| *Trypanosome brucei* | Tb | TriTrypDB | TriTrypDB |
| *Trypanosoma cruzi* | Tc | TriTrypDB | TriTrypDB |
| *Theileria parva* | Tpa | NCBI | CryptoDB |
| *Thalassiosira pseudonana* | Tps | JGI | JGI |
| *Tetrahymena thermophila* | Tth | TGD | TGD |
| *Thecamonas trahens* | Ttr | Broad | Broad |
| *Trichomonas vaginalis* | Tv | TrichDB | TrichDB |
| *Vitrella brassicaformis* | Vb | CryptoDB | CryptoDB |
| *Xenopus tropicalis* | Xt | JGI | NCBI |
